# Applications of Nisin and EDTA in Food Packaging for Improving Fabricated Chitosan-Polylactate Plastic Film Performance and Fish Fillet Preservation

**DOI:** 10.3390/membranes11110852

**Published:** 2021-11-04

**Authors:** Shun-Hsien Chang, Ying-Ju Chen, Hsiang-Jung Tseng, Hsin-I Hsiao, Huey-Jine Chai, Kuo-Chung Shang, Chorng-Liang Pan, Guo-Jane Tsai

**Affiliations:** 1 Institute of Food Safety and Risk Management, National Taiwan Ocean University, Keelung 202301, Taiwan; lewis@mail.ntou.edu.tw; 2 Department of Food Science, National Taiwan Ocean University, Keelung 202301, Taiwan; ingridswim@gmail.com (Y.-J.C.); hi.hsiao@mail.ntou.edu.tw (H.-I.H.); b0037@mail.ntou.edu.tw (C.-L.P.); 3 Research and Development Department, Plastic Industry Development Center, Taichung 40768, Taiwan; hunter0802@pidc.org.tw; 4 Seafood Technology Division, Fisheries Research Institute, Council of Agriculture, Keelung 202008, Taiwan; hjchai@mail.tfrin.gov.tw; 5 Department of Transportation Science, National Taiwan Ocean University, Keelung 202301, Taiwan; gordon@mail.ntou.edu.tw; 6 Center for Marine Bioenvironment and Biotechnology, National Taiwan Ocean University, Keelung 202301, Taiwan

**Keywords:** chitosan, PLA composite film, EDTA, nisin, antimicrobial activity, fish preservation

## Abstract

This study aimed to increase the antibacterial activity of chitosan-polylactic acid (PLA) composite film by adding nisin and ethylenediaminetetraacetic acid (EDTA). We evaluated the mechanical, physicochemical, and antibacterial properties of various PLA composite films, as well as the enhancement effect of PLA composite films with EDTA + nisin on the preservation of grouper fillets. Films of PLA alone, PLA plus chitosan (C5), PLA plus nisin + EDTA (EN2), and PLA plus chitosan plus nisin + EDTA (C5EN1 and C5EN2) were prepared. The addition of EDTA + nisin to the chitosan-PLA matrix significantly improved the antibacterial activity of the PLA composite film, with C5EN1 and C5EN2 films showing the highest antibacterial activity among the five films. Compared with the fish samples covered by C5, the counts of several microbial categories (i.e., mesophilic bacteria, psychrotrophic bacteria, coliforms, *Aeromonas*, *Pseudomonas*, and *Vibrio*) and total volatile basic nitrogen content in fish were significantly reduced in the samples covered by C5EN1. In addition, the counts of samples covered by C5EN1 or C5 were significantly lower compared to the uncovered and PLA film-covered samples.

## 1. Introduction

Aquatic products are susceptible to microbial deterioration. Every year, approximately 30 million tons of aquatic products are not properly preserved after being caught, leading to their deterioration [1]. Fish meat is more susceptible to spoilage than livestock meat owing to its higher content of water and free amino acid and lower content of connective tissue. Compared with poultry, various biochemical and enzymatic changes are triggered in fish muscles immediately after death, especially at incorrect processing temperatures. Both these changes and microbial activity cause the degradation of fish muscle [2]. Various fish preservation techniques are used to prevent the fish quality from deterioration and extend its shelf-life; these include the use of preservatives, the management of water activity and pH, and the combination of packaging and cold-chain transportation systems. [3].

Polylactic acid (PLA), which is biodegradable and biocompatible, has been applied in various fields as a packaging and supporting material [4]. However, owing to its heat sensitivity and poor tensile strength, PLA is frequently co-formulated with other flexible biopolymers, plasticizers, fibers, and nanofillers [5,6]. Several researchers have developed PLA composite films for food preservation. Talebi et al. [7] incorporated 1% spice essential oils (*Mentha piperita* and *Bunium percicum*) into the PLA film matrix, which strongly inhibited the growth of *Staphylococcus aureus*, *Enterobacteriaceae*, *Pseudomonas*, and lactic acid bacteria in ground beef and extended its shelf-life from 4 days to 7 days. Llana-Ruiz-Cabello et al. [8] used PLA films incorporating 2–6.5% of *Allium* herbal extracts to package ready-to-eat salads. All the extract-containing PLA films effectively suppressed enterobacterial growth in salad samples, with the greatest effect observed for film containing 6.5% extract. Yang et al. [9] developed biodegradable films based on PLA blended with poly(butylene succinate adipate) (PBSA) and carvacrol (CAR) for food preservation, in particular for aquatic products. The addition of CAR increased the mobility of PLA/PBSA chains and improved their flexibility and ductility. This CAR-added PLA/PBSA film effectively inhibited the growth of bacteria and extended the cold-storage shelf-life of salmon fillets by 3–4 days [10].

Chitosan, a polymer composed of glucosamine and *N*-acetylglucosamine from crustaceans, insects, and fungi, has strong antimicrobial activity and has been used in clinical, agricultural, and food products [11]. Various antibacterial films made from chitosan alone or chitosan with other bioactive agents, such as cumin [4], guava peel extract [12], and ginger oil [13], have been developed to extend the shelf-life of foods. However, owing to the high water permeability and low mechanical strength of chitosan films, chitosan must be combined with other less water-impermeable polymers to make biodegradable antibacterial films. Some types of chitosan-PLA films have been developed, including chitosan/poly (vinyl alcohol) PLA film [14] and chitosan/polyethylene glycol PLA film [15]. In our previous report, we prepared a chitosan-PLA composite film for the preservation of fish fillets [16]. As the mechanism of chitosan antimicrobial action is mediated through the electrostatic interaction between the protonated amine residues in the chitosan molecule and the negatively charged groups on the bacterial surface [12,17], the biocidal effect of chitosan film, as proposed by Zimet et al. [18], may be due to the release of protonated glucosamine fractions from the biopolymer. However, the larger molecular weight and poor water-solubility of the chitosan (220 kDa) used in our previous chitosan-PLA film [16] may have limited the release of glucosamine residues, and thus limited the preservative effect on grouper fillet at room temperature. Only low-temperature storage could clearly show the ability of this film to extend the shelf-life of grouper [16].

Nisin, a small antibacterial peptide (3500 Da) produced by *Lactococcus lactis*, is the most widely used bacteriocin in more than 48 countries and is approved by the USFDA. This peptide can inhibit the growth of a broad spectrum of gram-positive microorganisms, including *Listeria monocytogenes* and *Staphylococcus aureus*, and prevent spore germination [19]. It is well accepted that nisin exerts better potency against Gram-positive [G(+)] bacteria than Gram-negative [G(−)] bacteria as the cell wall of gram-positive bacteria is highly negatively charged. The electrostatic interaction between the positive charges in nisin and the negatively charged cell surface allows the nisin peptide to attach to the bacterial cell membrane [20], and the hydrophobic amino acid of the peptide inserts deeper into the bacterial cell, changing the permeability of the bacterial cell membrane and inducing bacterial death [21]. Ethylene diamine tetraacetic acid (EDTA) is a safe, economical metal chelator that sequesters divalent cations (notably Ca^2+^ and Mg^2+^) that contribute to the stability of the outer membrane of gram-negative bacteria [17]. Therefore, EDTA improves the activity of nisin against gram-negative bacteria, including *E. coli* O157:H7 and *S. typhimurium* in in vitro tests [22].

The chitosan-PLA composite film we prepared previously had a much weaker effect at 25 °C than at 4 °C on the preservation of grouper fillet [16]. In the present study, we aimed to improve the antibacterial function of a chitosan-PLA composite film through the incorporation of the low-molecular-weight antibacterial agents of nisin and EDTA. Films of PLA alone, PLA plus chitosan, PLA plus nisin + EDTA, and PLA plus chitosan plus nisin + EDTA were prepared. Their mechanical, physicochemical, and antibacterial properties were determined. Their application for grouper fillet preservation was also evaluated.

## 2. Materials and Methods

### 2.1. Bacterial Strains and Chemicals

*Escherichia coli* BCRC 11,634 and *Staphylococcus aureus* BCRC 10,451 were purchased from the Biosources Collection and Research Center (Hsinchu, Taiwan). EDTA, sodium bicarbonate (NaHCO_3_), and nisin (10^6^ IU) were obtained from Sigma Chemical Co. (Gillingham, UK). Chitosan powder was obtained from Applied Chemical Co., Ltd. (Kaohsiung, Taiwan). Bacto agar, nutrient broth (NB), nutrient agar, plate count agar (PCA), *Pseudomonas* isolation agar, starch ampicillin agar (SAI), thiosulphate-citrate-bile salts-sucrose, tryptic soy broth, and violet red bile agar were supplied by Becton Dickinson (Sparks, MD, USA). Polylactic acid (PLA, manufactured by Natureworks@ (4032D), Minnetonka, MA, USA) had a weight-average molecular weight (Mw) of 1.96 × 10^4^ Da, as determined by gel permeation chromatography.

### 2.2. Antibacterial Activity of Chitosan, Nisin, and EDTA

*E. coli* BCRC 11,634 and *S. aureus* BCRC 10,451 were incubated in NB at 37 °C for 24 h for use as the bacterial cultures. The sterile stock solutions of 1M EDTE-2Na, 10% (*w/v*) nisin, and 1% (*w*/*v*) chitosan were prepared based on the methods of Ukuku and Fett [23], Hoffman et al. [24], and Chang et al. [16], respectively. To vials containing 10 mL NB, different volumes of chitosan stock solution, nisin stock solution, and EDTA-2Na stock solution were added to prepare final concentrations of 5 ppm chitosan, 20 mM EDTA-2Na, and 0–75 µg/mL nisin in NB, respectively. After inoculation of bacterial cultures with an initial cell density of 10^6^ CFU/mL into chitosan/EDTA/nisin-containing NB and incubation at 37 °C for 24 h, the viable cells were measured using a plate counting method.

### 2.3. Film Preparation

Film preparation was based on the method of Chang et al. [16]. The films containing PLA alone, PLA + 0.5% (*w*/*w*) chitosan (abbreviated as C5), PLA + 20 mM EDTA + 0.02% (*w*/*w*) nisin (abbreviated as EN2), and PLA + 0.5% chitosan + 20 mM EDTA + 0.01 %−0.02% nisin (abbreviated as C5EN1 and C5EN2) were manufactured by Plastics Industry Development Center (Taichung, Taiwan, ROC). The base mixture without chitosan was used as the PLA control. Extrusion on a casting laminating machine (SHFV-QA16010, HsinPow Machinery Co. Ltd., Tainan, Taiwan) was used to produce the chitosan-EDTA-nisin-PLA composite films, which were finally trimmed into film rolls of 30 cm in width and 0.04 mm in thickness.

### 2.4. Antimicrobial Activity of Films

The antimicrobial activity of film was measured in accordance with the protocol of Chang et al. [16]. In brief, the diluted cultures (2.5–10 × 10^5^ CFU/mL) of *E. coli* BCRC 11,634 and *S. aureus* BCRC 10,451 incubated in NB at 37 °C for 24 h were used as the bacterial cultures. In total, 0.4 mL of the diluted culture was added to the test samples of PLA, C5, C5EN1 or C5EN2, and EN2 films, which had been sterilized in UV-light for 24 h. After incubation at 37 °C for 24 h, the test films were rinsed with 10 mL of SCDLP broth. The viable bacterial count in the washed SCDLP broth was measured by counting the colonies on the PCA plate. The tests were conducted in triplicate.

### 2.5. Application on the Preservation of Fish Fillet

Based on the method of Chang et al. [16], fresh grouper (*Epinephelus fuscoguttatus* × *Epinephelus lanceolatus*) fillets were cut into smaller fillets and the upper and the lower surfaces of the fillets were covered with the test films; fish fillet with no covering was used as the control. All samples were placed in dishes and stored at 4 °C or 25 °C. Five samples were removed from each storage condition: two were used for the measurement of total volatile basic nitrogen (TVBN) content using Conway’s method [25]; the remaining three were used for pH measurements and microbial analysis. Surface plating counts were adopted by spreading decimal diluents (100 µL) on various media and the incubation conditions used were as described in the method of Chang et al. [16].

### 2.6. Mechanical and Physical Properties

#### 2.6.1. Mechanical Properties

The mechanical properties were investigated based on the determination of the tensile strength and elongation at break of the films in accordance with the ASTM D882 standard method [26]. The tensile strength and elongation at break were calculated from the stress-strain curves. The tear strength was measured in accordance with ASTM D1938 method.

#### 2.6.2. Water Vapor Transmission Rate

The test film was sealed over the top of a test tube containing anhydrous silica gel at 20 °C ± 2 °C [16]. The water vapor transmission rate (g mm/m^2^ day kPa) was calculated from the increase in the test tube weight over time after the transfer reach steady-state. The test was conducted in triplicate.

#### 2.6.3. Moisture Content

The moisture content of the film was calculated as the percentage reduction in the dry weight of the film reduction (50 mg) at 105 °C until a constant weight was reached [16]. The test was conducted in triplicate.

#### 2.6.4. Overall Migration Test

Based on the method of Tovar et al. [27], but with some modifications, three aqueous simulants of distilled water, 10% aqueous ethanol (*v*/*v*), and 3% aqueous acetic acid (*w/v*) were used. A piece of the test film (3 cm × 4 cm) and 20 mL of simulant were placed in a glass vial at 40 °C for 10 days. After the film was removed, the simulant was vacuum-evaporated to dryness and the solid residue was gravimetrically analyzed. Six replicates were performed for each film and each stimulant.

### 2.7. Statistical Analysis

All data were analyzed statistically using repeated-measure and one-way analysis of variance (ANOVA), and multiple comparisons between treatment means were completed by Duncan’s tests. All experiments were performed in triplicate, and an evaluation of the statistical significance at *p* < 0.05 was performed using SPSS 16.0 software (SPSS Inc., Chicago, IL, USA). The data were expressed as mean values with standard deviation (mean ± SD).

## 3. Results and Discussion

### 3.1. Antimicrobial Activity of Chitosan, EDTA, Nisin, and Combined Films

**Figure 1:**
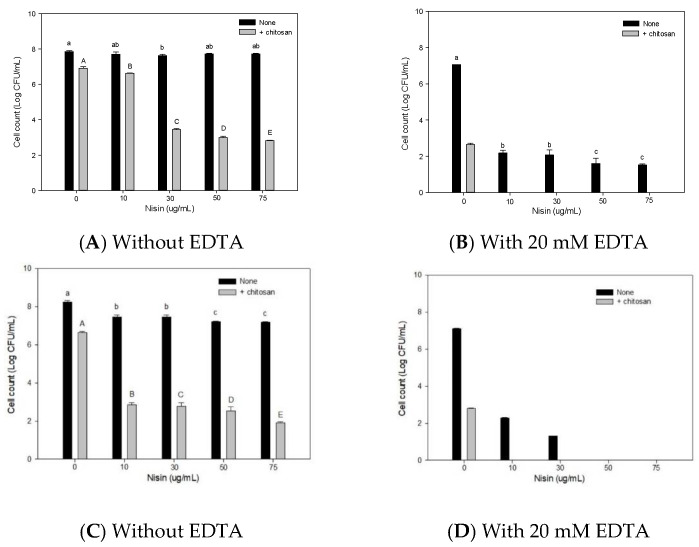
Antibacterial activity of chitosan (5 μg/mL) in NB broth containing various concentrations of nisin without EDTA (**A**,**C**) and with EDTA (20 mM) (**B**,**D**) against *Escherichia coli* BCRC11634 (**A**,**B**) and *Staphylococcus aureus* BCRC 10451(**C**,**D**).

Nisin is the most widely used bacteriocin in the world. It has a strong inhibitory effect on gram-positive bacteria and a weaker inhibitory effect on Gram-negative bacteria [20]. The metal chelator, EDTA, can increase the effect of nisin on Gram-negative bacteria [28]. Therefore, the antibacterial activity of the combination of chitosan, nisin, and EDTA was first evaluated in vitro, and then PLA composite membranes were prepared. In the absence of EDTA (A, C) or the presence of 20 mM EDTA (B, D), the antibacterial effects of various concentrations of nisin (with/without 5 µg/mL chitosan) in NB broth against the G(−) (bacterium *E. coli* BCRC11634 (A, B) and the G(+) bacterium *S. aureus* BCRC 10,451 (C, D) are shown in Figure 1. Nisin alone (10–75 µg/mL), without 5 µg/mL chitosan, had almost no activity against *E. coli*, whereas chitosan alone at 5 µg/mL had some activity against *E. coli*. The antibacterial activity of the combination of chitosan (5 µg/mL) and nisin gradually increased as the nisin concentration increased (Figure 1A). Compared with the survival (8 log CFU/mL) of the control (without chitosan and nisin) in Figure 1A, the survival in the broth containing only 20 mM EDTA in Figure 1B was reduced by approximately 0.9 log CFU/mL. The survival in 20 mM EDTA plus 5 µg/mL chitosan and 20 mM EDTA plus various concentrations of nisin were greatly reduced (Figure 1B), compared with the survival of 5 µg/mL chitosan alone or nisin without EDTA in Figure 1A, respectively. This demonstrated that EDTA could enhance the bactericidal effect of chitosan and nisin on *E. coli.* In addition to the inhibitory effect of nisin alone on *S. aureus* (Figure 1C), 20 mM EDTA similarly increased the inhibitory effect of chitosan and nisin on *S. aureus* (Figure 1D). In brief, 20 mM EDTA could significantly enhance the antibacterial activity of both chitosan and nisin against *E. coli* and *S. aureus*. The bactericidal effect of chitosan was greatly increased by the combination of EDTA and nisin. Similarly, Hui et al. [29] showed that nisin combined with chitosan treatment had stronger antibacterial activity and provided better quality for yellow croaker during storage.

### 3.2. Mechanical and Physical Properties of Films

**Figure 2:**
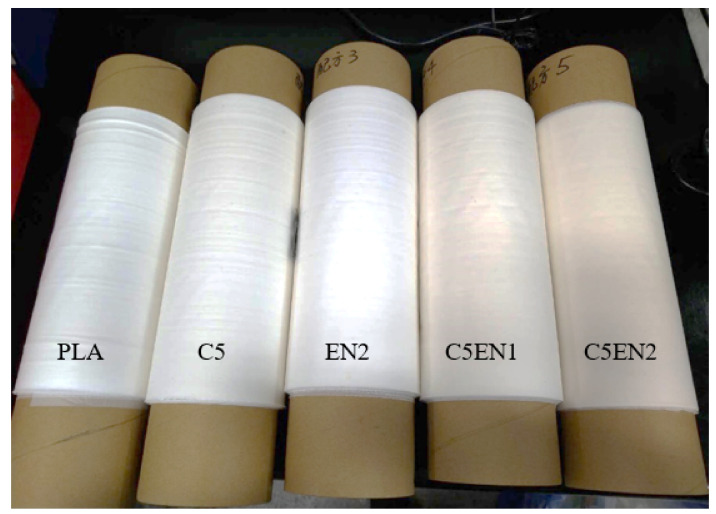
Appearance of PLA, C5, EN2, C5EN1, and C5EN2 films. (Film thickness: 30 μm).

The five films ((PLA (as control), C5 (PLA + 0.5% of chitosan), EN2 (PLA + 20 mM EDTA + 0.02% nisin), C5EN1 (PLA + 0.5% chitosan + 20 mM EDTA + 0.01% nisin), and C5EN2 (PLA + 0.5% chitosan + 20 mM EDTA + 0.02% nisin)) were prepared and their visual appearances are shown in Figure 2. Compared with the PLA and EN2 films, the C5, C5EN1, and C5EN2 films were comparable yellowish, which may be due to chitosan’s partial miscibility affecting the color of the continuous matrix [30].

For polymeric films to provide sufficient physical protection to maintain food integrity, adequate mechanical properties are very important [31]. Therefore, the tensile strength, elongation at break, and tear strength were investigated as a function of chitosan and/or EDTA + nisin incorporation in the PLA matrix; the results are shown in Table 1.

The inclusion of either chitosan or EDTA + nisin into the PLA matrix greatly reduced the tensile strength and elongation at break [in both machine direction (MD) and transverse direction (TD)], as shown in C5 and EN2. However, as shown in C5EN1 and C5EN2, the tensile strength and elongation at break after the inclusion of chitosan and EDTA + nisin were not significantly different from the C5 and EN2. Bonilla et al. [30] proposed that chitosan particles may cause irregularities and discontinuities in the oriented PLA matrix, which can result in the decrease of tensile strength. A similar explanation may apply for the reduction resulting from the addition of EDTA + nisin to the PLA matrix on the tensile strength and elongation at break. However, adding either chitosan or EDTA + nisin into PLA matrix greatly increased the tear strength of the composite PLA films, as shown in EN2 and C5. When these two components are both added to the PLA matrix, the increase in the tear strength was slightly reduced, as shown with C5EN1 and C5EN2. Although we are currently unable to provide an explanation for this, the helical configuration of chitosan [32] and nisin [33], and the electronic attraction between chitosan-PLA and EDTA-PLA may help increase the tear strength (gf) of the film. When both chitosan and EDTA + nisin are added to PLA, the charge interaction between chitosan and EDTA may reduce the electronic force between PLA and each component. Therefore, the tear strength for C5EN1 and C5EN2 were lower than that of EN2 and C5.

Water plays a central role in various chemical and microbial spoilage reactions in food. With a suitable packaging film that can reduce water vapor transmission rate, the free water content of food can be gradually reduced, thereby extending the shelf-life [7]. The water vapor transmission rate and moisture content of PLA, EN2, C5, C5EN1, and C5EN2 films are shown in Table 2. Owing to the hydrophilicity of chitosan [34], the water vapor transmission rate and moisture content of chitosan-containing PLA composite films, such as C5, C5EN1, and C5EN2 were significantly increased. Similarly, the hydrophilic EDTA + nisin in the PLA matrix also increased the water vapor transmission rate and moisture content of the film. Consequently, C5EN2 had the highest transmission rate (0.85 ± 0.12 g mm/m^2^ day kPa) and moisture content (1.68% ± 0.78%).

In this study, chitosan and/or EDTA + nisin was directly mixed with the PLA matrix to prepare a PLA composite film, which was used as a packaging material in contact with food. To comply with food contact material legislation, the total mass of packaging compounds released into food or food simulants was monitored. Three aqueous simulants (distilled water, 10% ethanol, and 3% acetic acid) were used, and the harshest conditions (40 °C for 10 days) were used, in accordance with the 97/48/CE [35], directive to represent the worst-case food packaging material. As shown in Table 3, the total migration of the five test films to the three simulants was very low. Even the highest migration mass, of C5EN2 (3.04 ± 0.18 μg/dm^2^) in 3% acetic acid, was still well below the 10 μg/dm^2^ limit set by the European Commission [36]. This confirmed the safety of all tested films.

### 3.3. Antibacterial Activity of Films

**Figure 3:**
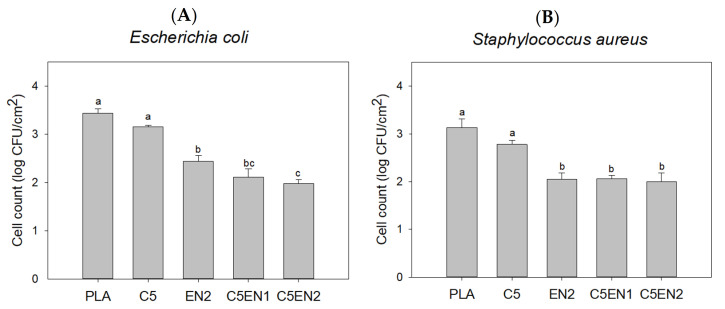
Antibacterial activity of chitosan, EDTA and nisin composite PLA films against *Escherichia coli* BCRC11634 (**A**) and *Staphylococcus aureus* BCRC10451 (**B**). PLA, PLA film as control; C5, PLA + 0.5% chitosan; EN2, PLA + 20 mM EDTA + 0.02% nisin; C5EN1, PLA + 0.5% chitosan + 20 mM EDTA + 0.01% nisin; C5EN2, PLA + 0.5% chitosan + 20 mM EDTA + 0.02% nisin. Data are presented as mean ± SD (n = 3). Different superscript in the same column indicates significant difference (*p* < 0.05).

The antibacterial activity of PLA and various PLA composite films against *E.* *coli* (A) and *S.* *aureus* (B) is shown in Figure 3. There was no significant difference in the survival of *E. coli* with the PLA and C5 films. Compared with PLA, the survival of *E. coli* in the EN2, C5EN1, and C5EN2 were significantly reduced (Figure 3A). Similarly, there was no significant difference in the survival of *S. aureus* with the PLA and C5 films, whereas the survival of *S. aureus* with the EN2, C5EN1, and C5EN2 films were significantly reduced by 1.2 log CFU/cm^2^ compared with that of PLA (Figure 3B).

Antibacterial materials can be added directly to food formulations or slowly releasing from packaging materials. The application of the antibacterial film allows the antibacterial agent to migrate to the surface of the film and confers a sustained antibacterial effect to the food over a prolonged period [37]. In addition, antibacterial agents in film may be protected from inactivation by food enzymes [38]. Salmaso et al. [39] observed that nisin-loaded PLA materials prolonged nisin activity by up to 40 days whereas the free nisin samples displayed antimicrobial activity for only 7 days. Compared with the molecular size of chitosan, the molecular weight of nisin and EDTA is smaller; consequently, it can migrate more easily in the PLA film. Therefore, the chitosan-PLA composite film containing nisin + EDTA in this study, such as C5EN1 and C5EN2, will have higher antibacterial activity than the chitosan-PLA composite film (C5). In short, C5EN1 and C5EN2 have similar mechanical properties and antibacterial activity. Therefore, we chose PLA, C5, EN2, and C5EN1 for the subsequent fish fillet preservation test.

### 3.4. Application of the PLA Composite Film for Preservation of Fish Fillets

**Figure 4:**
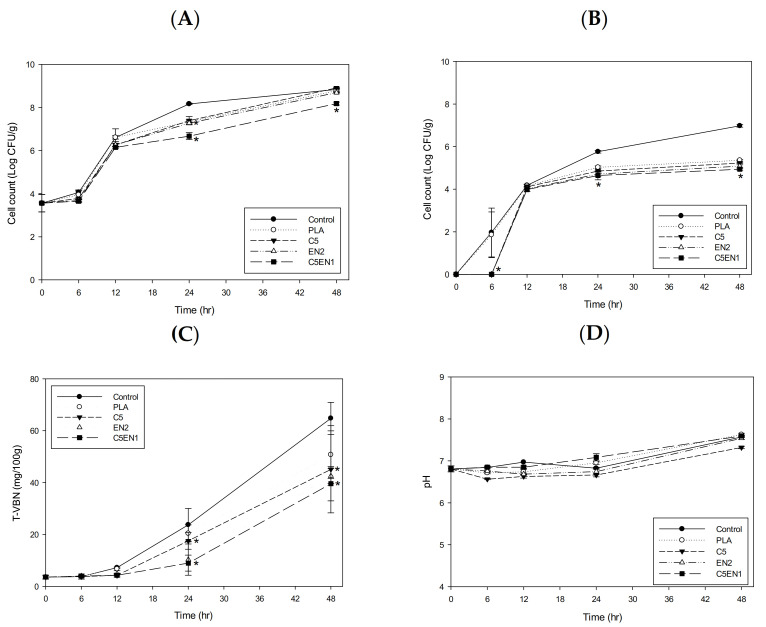
Changes in mesophilic bacteria count (**A**), psychrotrophic bacteria count (**B**), total volatile basic nitrogen content (TVBN) (**C**) and pH value (**D**) of fish fillet (*Epinephelus fuscoguttatus* x *Epinephelus lanceolatus*) covered with PLA, C5, EN2, or C5EN1 film during 25 °C storage for 48 h. PLA, PLA film as control; C5, PLA + 0.5% chitosan; EN2, PLA + 20 mM EDTA + 0.02% nisin; C5EN1, PLA + 0.5%chitosan + 20 mM EDTA + 0.01% nisin. Data are presented as mean ± SD (n = 3). * means significantly different, compared to control (*p* < 0.05).

In our previous report, the chitosan-PLA composite film showed a better preservation effect on fish fillets at 4 °C than at 25 °C [16]. In this study, we attempted to increase the antibacterial activity of the chitosan-PLA film through the addition of the lower molecular weight antibacterial agents of nisin and EDTA. After confirming the enhancement effect of EDTA + nisin on chitosan in vitro (Figure 1) and in the chitosan-PLA composite membrane (Figure 3), we used our previously reported method [16] to evaluate the potential enhancement of the preservation of fish fillets. As shown in Figure 4A, after storage at 25 °C for 6 h, C5EN1 effectively inhibited the increase in mesophilic bacteria count in fish fillets. After 24 h, the mesophilic bacteria count of the fish fillets covered by C5EN1 was significantly lower than that of the fish fillets covered by the other three types of film, and were significantly lower than the uncovered fish fillets (Figure 4A). A similar suppression of the increase in the psychrotrophic bacteria count of fish fillets covered with C5EN1 was also observed over the first 6 h of storage (Figure 4B). However, the psychrotrophic bacteria counts in the fish fillets covered with the different films were similar, and all were significantly lower than the uncovered fish fillets during the storage periods of 24 and 48 h (Figure 4B).

The TVBN is a quantitative parameter that reflects the degree of fish spoilage [40]. As shown in Figure 4C, the TVBN value of the fillets was highest for PLA, followed by C5, EN2, and C5EN1 in order (i.e., lowest in the C5EN1-covered fillets), and all values were significantly lower than that for the uncovered fillets. The pH values for all groups were gradually increased from 6.79 to 7.02–7.18. There was no difference in pH between the test groups (Figure 4D). Hui et al. [30] reported that pH is not reliable as an indicator of fish quality evaluation. Due to the high buffering capacity of the protein in fish, it can be observed that the pH difference between treatments is much smaller. Similar results for the pH changing profiles of tiger tooth croaker [41] and catfish fillet [42] during storage were obtained.

**Figure 5:**
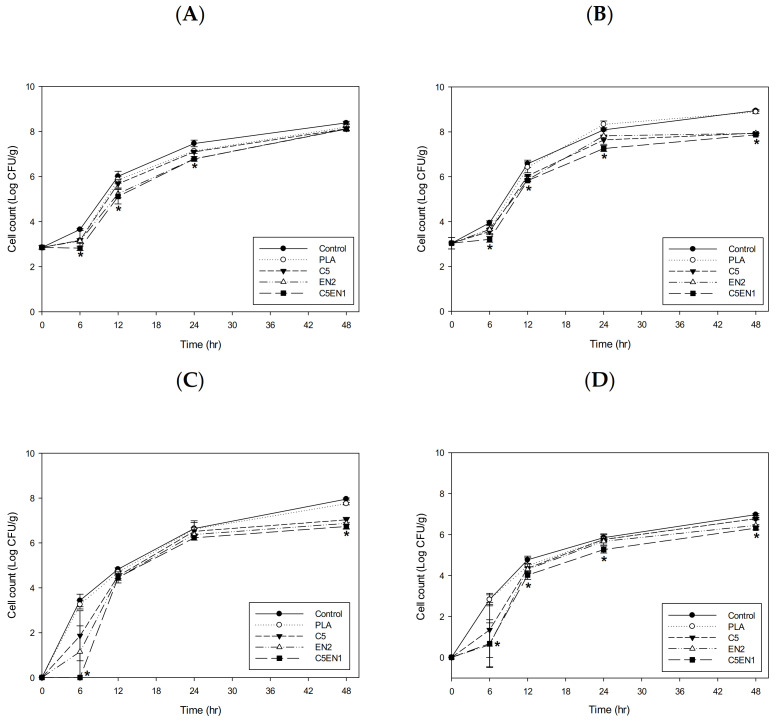
Changes in cell counts of *coliforms* (**A**), *Aeromonas* (**B**), *Pseudomonas* (**C**) and *Vibrio* (**D**) in fish fillet (*Epinephelus fuscoguttatus* × *Epinephelus lanceolatus*) covered with PLA, C5, EN2, or C5EN1 film during storage at 25 °C for 48 h. PLA, PLA film as control; C5, PLA + 0.5% chitosan; EN2, PLA + 20 mM EDTA + 0.02% nisin; C5EN1, PLA + 0.5%chitosan + 20 mM EDTA + 0.01%nisin. Data are presented as mean ± SD (n = 3). * means significantly different, compared to control (*p* < 0.05).

The cell counts of different microbiomes in fish fillets covered with the test films and stored at 25 °C for 48 h are shown in Figure 5. The numbers of coliforms (Figure 5A), Aeromonas (Figure 5B), Pseudomonas (Figure 5C), and Vibrio (Figure 5D) in the C5EN1-covered samples did not increase until after storage for 6 h. After storage for 24 h, the counts for these different microbial groups in the C5EN1-covered fillets were significantly lower than the C5-covered fillets, both of which were significantly lower than the control group (uncovered fillets) (Figure 5).

**Figure 6:**
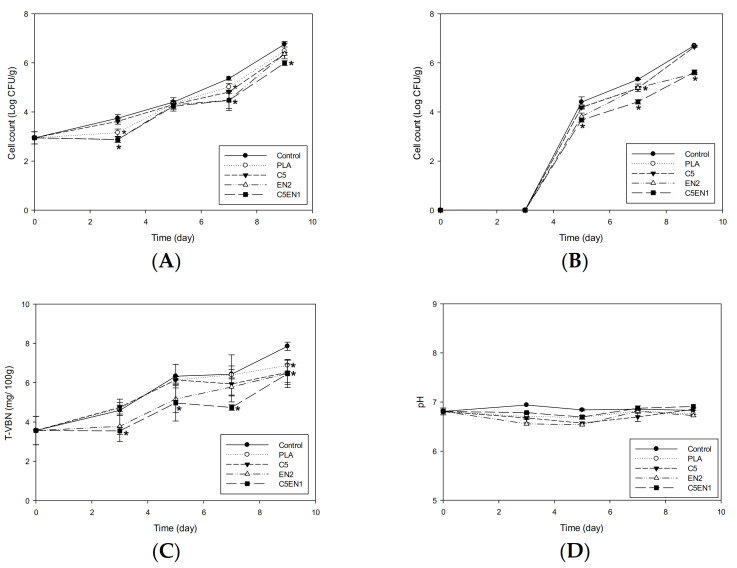
Changes in mesophilic count (**A**), psychrotrophic count (**B**), TVBN content (**C**) and pH value (**D**) of fish fillet (*Epinephelus fuscoguttatus* × *Epinephelus lanceolatus*) covered with PLA, C5, EN2, or C5EN1 film during storage at 4 °C. PLA, PLA film as control; C5, PLA + 0.5% chitosan; EN2, PLA + 20 mM EDTA + 0.02% nisin; C5EN1, PLA + 0.5%chitosan + 20 mM EDTA + 0.01% nisin. Data are presented as mean ± SD (n = 3). * means significantly different, compared to control (*p* < 0.05).

The changes in the mesophilic bacteria count, psychrotrophic bacteria count, TVBN, and pH of fish fillet that were not covered with film (the control samples) or were covered with PLA, C5, EN2, and C5EN1-PLA composite films during storage at 4 °C are shown in Figure 6. Unlike the results from storage at 25 °C, the EN2 and C5EN1 films effectively inhibited the increase in the mesophilic bacteria count in the fish fillets over the first 3 days of storage at 4 °C. After storage for 7 days, the number of mesophilic bacteria in the C5EN1-covered fillets was significantly lower than that of the C5-covered fillets, and both were significantly lower than the control. After 9 days of storage, the mesophilic bacteria count in the control group and the samples covered by PLA-, C5-, or EN2 exceeded the 6 log CFU/g (control limit), whereas the bacteria count in the fillets covered by C5EN1 was still below this control limit (Figure 6A). Although no psychrotrophic bacteria growth was observed in any group during the first three days of storage, in the samples covered with various test films, the influence of the film on the number of psychrotrophic bacteria and mesophilic bacteria was similar. After 9 days of storage, the psychrotrophic bacteria count of the samples covered by C5EN1 was significantly lower than that of the samples covered by C5, and both were significantly lower than the control samples (Figure 6B). After 9 days of storage at 4 °C, the TVBN content of all the tested fish fillets was below 10 mg/100 g. The C5EN1 sample was the lowest (7 mg/100 g), which was far below the control limit for raw fish fillet (25 mg/100 g) (Figure 6C). After 9 days of storage, no significant differences were observed in the pH values of all groups (Figure 6D). Although chitosan coating combined with glycerol monolaurate had been shown to inhibit microbial spoilage and delay the formation of alkaline compounds during the refrigerated storage of grass carp fillets (GCFs) [43]. However, there are no significant differences among all the treated samples (*p* > 0.05).

**Figure 7:**
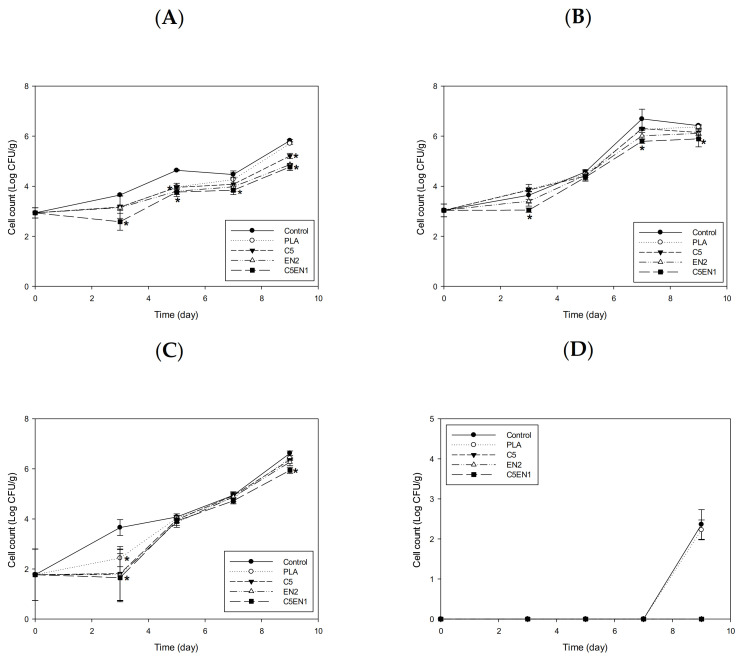
Changes in cell counts of *Escherichia* (**A**), *Aeromonas* (**B**), *Pseudomonas* (**C**), *Vibrio* (**D**) in fish fillet (*Epinephelus fuscoguttatus* × *Epinephelus lanceolatus*) covered with PLA, C5, EN2, or C5EN1 film during storage at 4 °C. PLA, PLA film as control; C5, PLA + 0.5% chitosan; EN2, PLA + 20 mM EDTA + 0.02% nisin; C5EN1, PLA + 0.5%chitosan + 20 mM EDTA+0.01% nisin. Data are presented as mean ± SD (n = 3). * means significantly different, compared to control (*p* < 0.05).

The changes in cell counts of special microbial groups of *Escherichia* (A), *Aeromonas* (B), *Pseudomonas* (C), and *Vibrio* (D) in fish fillets covered by the PLA, C5, EN2, or C5EN1 films during storage at 4 °C are shown in Figure 7. Over the first 3 days, covering the fish fillets with the C5, EN2, or C5EN1 films effectively inhibited the increase in the numbers of coliforms (Figure 7A), *Aeromonas* (Figure 7B), and *Pseudomonas* (Figure 7C). After 9 days of storage, the number of coliforms (Figure 7A), *Aeromonas* (Figure 7B), and *Pseudomonas* (Figure 7C) in the samples covered by the C5EN1 film was significantly lower than that of the samples covered by C5, and they were all significantly lower than those of the control (uncovered) fillets or PLA-covered fillets. The *Vibrio* count in the fish fillets covered with the C5, EN2, or C5EN1 films was not detectable during storage at 4 °C, whereas the the *Vibrio* count in the control and PLA samples was approximately 2 log CFU/g after storage at 4 °C for 9 days (Figure 7D).

Nisin is the most widely used bacteriocin. It is produced by *L.* *lactis*, consists of 34 amino acids, and has a molecular weight of 3500 Da. Although nisin has broad spectrum effects on various G(+) bacteria, such as *Bacillus*, *Clostridium*, *Lactococcus*, *Listeria*, *Mycobacterium*, *Staphylococcus*, and *Streptococcus*, it cannot inhibit G(−) bacteria owing to the presence of outer membrane [44]. Belfiore, et al. [45] demonstrated that the metal ion chelating agent EDTA could bind to the Mg^2+^ and Ca^2+^ ions in cell wall (G(+) bacteria) or in the outer membrane (G(−) bacteria), thereby destroying the stable cell structure, which favors the pore formation on cell surface by nisin. Therefore, EDTA could increase the inhibitory effect of nisin on both G(+) and G(−) bacteria [20,46]. Nisin has acid and thermal stability, but it is sensitive to various proteases [28,47]. Therefore, nisin in the membrane can protect its activity from degradation by food enzymes [38].

Through the results of this study, we have confirmed that the addition of nisin + EDTA can greatly enhance the antibacterial activity of chitosan in solution (Figure 1) or in a chitosan-PLA composite film (Figure 3). The addition of nisin + EDTA to the chitosan-PLA matrix significantly enhanced the antibacterial activity of the composite membranes, such as C5EN1 and C5EN2. Therefore, compared with C5 (the chitosan-PLA) film, the C5EN1 (chitosan-PLA with nisin + EDTA) film had a better preservative efficacy on fish fillets stored at 25 °C (Figure 4 and Figure 5) or 4 °C (Figure 6 and Figure 7). This was consistent with some other reports. Bhatia and Bharti [48] proposed that nisin + EDTA had a partial synergistic effect on the antibacterial activities of chitosan-starch packaging film. Divsalar, et al. [49] fabricated a composite film containing chitosan, cellulose, and nisin for use as antimicrobial packaging for ultra-filtered cheese. Pure chitosan-cellulose films do not exhibit antimicrobial activities against *L. monocytogenes*, whereas the film added with nisin resulted in significantly increased inhibition of *L. monocytogenes*.

In summary, the addition of nisin and EDTA greatly improved the antibacterial activity of chitosan in solution and in the chitosan-PLA film. The chitosan-PLA film with nisin + EDTA (C5EN1 in this study) significantly improved the preservation of fish fillets stored at 25 °C or 4 °C, and showed better performance than the chitosan-PLA film, which only had a significant effect for storage at 4 °C.

## 4. Conclusions

The addition of nisin and EDTA to chitosan-PLA matrix significantly increased the antibacterial activity of chitosan-PLA film and did not significantly affect the mechanical strength of chitosan-PLA film. Covering fish fillets with the nisin+EDTA added chitosan-PLA composite film (C5EN1 in this study) effectively reduced the mesophile, coliform, and spoilage bacteria counts, as well as the TVBN content during storage at 25 °C and 4 °C. Therefore, adding EDTA and nisin to the chitosan-PLA film is a promising solution to meet consumers’ demand for natural preservatives, and may be a breakthrough technology for preserving fresh food and extending shelf life.

## Figures and Tables

**Table 1:** Tensile strength, elongation at break, and tearing strength of PLA, EN2, C5, C5EN1, and C5EN2 films.

Film	Tensile Strength (kgf/cm^2^)		Elongation at Break (%)		Tearing Strength (gf)	
	MD	TD	MD	TD	MD	TD
PLA	140 ± 11 ^a^	104 ± 9 ^a^	215 ± 23 ^a^	51 ± 23 ^a^	110 ± 18 ^d^	264 ± 17 ^c^
EN2	63 ± 6 ^b^	35 ± 3 ^b^	48 ± 6 ^b^	16 ± 5 ^b^	407 ± 40 ^b^	367 ± 20 ^b^
C5	58 ± 8 ^b^	38 ± 2 ^b^	35 ± 5 ^b^	16 ± 1 ^b^	540 ± 30 ^a^	458 ± 40 ^a^
C5EN1	53 ± 7 ^b^	34 ± 2 ^b^	28 ± 4 ^b^	17 ±3 ^b^	346 ± 30 ^c^	214 ± 50 ^c^
C5EN2	47 ± 1 ^c^	32 ± 3 ^b^	29 ± 8 ^b^	16 ± 1 ^b^	387 ± 41 ^c^	387 ± 40 ^b^

MD, Machine Direction; TD, Transverse Direction; PLA, PLA film as control; C5, PLA + 0.5% chitosan; EN2, PLA + 20 mM EDTA + 0.02% nisin; C5EN1, PLA + 0.5% chitosan + 20 mM EDTA + 0.01% nisin; C5EN2, PLA + 0.5% chitosan + 20 mM EDTA + 0.02% nisin. Data are presented as mean ± SD (n = 3). Different superscript in the same column indicates significant difference (*p* < 0.05).

**Table 2:** Water vapor transmission rate and moisture content of PLA, C5, EN2, C5EN1, and C5EN2 films.

Film	Water Vapor Transmission Rate (g mm/m^2^ day kPa)	Moisture Content (%)
PLA	0.52 ± 0.12 ^c^	0.28 ± 0.23 ^d^
EN2	0.59 ± 0.05 ^b,c^	0.52 ± 0.43 ^c^
C5	0.64 ± 0.14 ^b^	1.27 ± 0.02 ^b^
C5EN1	0.68 ± 0.09 ^b^	1.01 ± 0.15 ^b^
C5EN2	0.85 ± 0.12 ^a^	1.68 ± 0.48 ^a^

MD, Machine Direction; TD, Transverse Direction; PLA, PLA film as control; C5, PLA + 0.5% chitosan; EN2, PLA + 20 mM EDTA+ 0.02% nisin; C5EN1, PLA + 0.5% chitosan + 20 mM EDTA + 0.01% nisin; C5EN2, PLA + 0.5% chitosan + 20 mM EDTA + 0.02% nisin. Data are presented as mean ± SD (n = 3). Different superscript in the same column indicates significant difference (*p* < 0.05).

**Table 3:** Overall migration mass of PLA and PLA composite films into various food simulants.

Simulants	Over Migration Mass (μg/dm^2^)				
	PLA	C5	EN2	C5EN1	C5EN2
Water	0.00 ± 0.00	0.00 ± 0.00	0.00 ± 0.00	0.00 ± 0.00	0.00 ± 0.00
10% Ethanol	0.00 ± 0.00 ^d^	0.25 ± 0.08 ^c^	0.37 ± 0.06 ^c^	0.53 ± 0.13 ^b^	0.79 ± 0.06 ^a^
3% Acetic acid	0.50 ± 0.24 ^c^	0.69 ± 0.13 ^c^	0.92 ± 0.30 ^c^	2.00 ± 0.35 ^b^	3.04 ± 0.18 ^a^

PLA, PLA film as control; C5, PLA + 0.5% chitosan; EN2, PLA + 20 mM EDTA + 0.02% nisin; C5EN1, PLA + 0.5% chitosan + 20 mM EDTA + 0.01% nisin; C5EN2, PLA + 0.5% chitosan + 20 mM EDTA + 0.02% nisin. Data are presented as mean ± SD (n = 3). Different superscript in the same column indicates significant difference (*p* < 0.05).

## Data Availability

The data used to support the findings of this study are available from the corresponding author upon request.

## References

[1] Nellemann C., MacDevette M. (2009). The Environmental Food Crisis: The Environment’s Role in Averting Future Food Crises: A UNEP Rapid Response Assessment.

[2] Amos B., Sector F., Einarsson H., Eythorsdottir A. (2007). Analysis of Quality Deterioration at Critical Steps/Points in Fish Handling in Uganda and Iceland and Suggestions for Improvement.

[3] Giannakourou M., Tsironi T. (2021). Application of Processing and Packaging Hurdles for Fresh-Cut Fruits and Vegetables Preservation. Foods.

[4] Avinc O., Khoddami A. (2010). Overview of Poly(lactic acid) (PLA) fibre. Fibre Chem..

[5] Gahleitner M., Grein C., Kheirandish S., Wolfschwenger J. (2011). Nucleation of Polypropylene Homo- and Copolymers. Int. Polym. Process..

[6] Xu H., Xie L., Chen J.-B., Jiang X., Hsiao B.S., Zhong G.-J., Fu Q., Li Z.-M. (2014). Strong and tough micro/nanostructured poly(lactic acid) by mimicking the multifunctional hierarchy of shell. Mater. Horizons.

[7] Talebi F., Misaghi A., Khanjari A., Kamkar A., Gandomi H., Rezaeigolestani M. (2018). Incorporation of spice essential oils into poly-lactic acid film matrix with the aim of extending microbiological and sensorial shelf life of ground beef. LWT.

[8] Llana-Ruiz-Cabello M., Pichardo S., Baños A., Núñez C., Bermúdez J., Guillamón E., Aucejo S., Cameán A. (2015). Characterisation and evaluation of PLA films containing an extract of Allium spp. to be used in the packaging of ready-to-eat salads under controlled atmospheres. LWT Food Sci. Technol..

[9] Yang C., Tang H., Wang Y., Liu Y., Wang J., Shi W., Li L. (2019). Development of PLA-PBSA based biodegradable active film and its application to salmon slices. Food Packag. Shelf Life.

[10] Yu Z., Li B., Chu J., Zhang P. (2018). Silica in situ enhanced PVA/chitosan biodegradable films for food packages. Carbohydr. Polym..

[11] Bautista-Baños S., Romanazzi G., Jiménez-Aparicio A. (2016). Chitosan in the Preservation of Agricultural Commodities.

[12] Yuan G., Lv H., Tang W., Zhang X., Sun H. (2016). Effect of chitosan coating combined with pomegranate peel extract on the quality of Pacific white shrimp during iced storage. Food Control.

[13] Remya S., Mohan C., Bindu J., Sivaraman G.K., Venkateshwarlu G., Ravishankar C.N. (2016). Effect of chitosan based active packaging film on the keeping quality of chilled stored barracuda fish. J. Food Sci. Technol..

[14] Grande R., Carvalho A.J.F. (2011). Compatible Ternary Blends of Chitosan/poly(vinyl alcohol)/poly(lactic acid) Produced by Oil-in-Water Emulsion Processing. Biomacromolecules.

[15] Sébastien F., Stéphane G., Copinet A., Coma V. (2006). Novel biodegradable films made from chitosan and poly(lactic acid) with antifungal properties against mycotoxinogen strains. Carbohydr. Polym..

[16] Chang S.-H., Chen Y.-J., Tseng H.-J., Hsiao H.-I., Chai H.-J., Shang K.-C., Pan C.-L., Tsai G.-J. (2021). Antibacterial Activity of Chitosan–Polylactate Fabricated Plastic Film and Its Application on the Preservation of Fish Fillet. Polymers.

[17] Tsai G.-J., Su W.-H. (1999). Antibacterial Activity of Shrimp Chitosan against Escherichia coli. J. Food Prot..

[18] Zimet P., Mombru A.W., Mombru D., Castro A., Villanueva J.P., Pardo H., Rufo C. (2019). Physico-chemical and antilisterial properties of nisin-incorporated chitosan/carboxymethyl chitosan films. Carbohydr. Polym..

[19] Yang S.-C., Lin C.-H., Sung C.T., Fang J.-Y. (2014). Corrigendum: Antibacterial activities of bacteriocins: Application in foods and pharmaceuticals. Front. Microbiol..

[20] Cole J.N., Nizet V. (2016). Bacterial Evasion of Host Antimicrobial Peptide Defenses. Microbiol. Spectr..

[21] Lei J., Sun L., Huang S., Zhu C., Li P., He J., Mackey V., Coy D.H., He Q. (2019). The antimicrobial peptides and their potential clinical applications. Am. J. Transl. Res..

[22] Tsai G.-J., Su W.-H., Chen H.-C., Pan C.-L. (2002). Antimicrobial activity of shrimp chitin and chitosan from different treatments and applications of fish preservation. Fish. Sci..

[23] Ukuku D.O., Fett W.F. (2004). Effect of nisin in combination with EDTA, sodium lactate, and potassium sorbate for reducing Salmonella on whole and fresh-cut cantaloupe. J. Food Prot..

[24] Hoffman K.L., Han I.Y., Dawson P.L. (2001). Antimicrobial Effects of Corn Zein Films Impregnated with Nisin, Lauric Acid, and EDTA. J. Food Prot..

[25] Conway E.J. (1947). Microdiffusion analysis and volumetric error. Microdiffusion Analysis and Volumetric Error.

[26] ASTM Subcommittee (2002). Standard Test Method for Tensile Properties of Thin Plastic Sheeting-D882–02. Annual Book of ASTM Standards.

[27] Tovar L., Salafranca J., Sánchez C., Nerín C. (2005). Migration studies to assess the safety in use of a new antioxidant active packaging. J. Agric. Food Chem..

[28] Khan A., Vu K.D., Riedl B., Lacroix M. (2015). Optimization of the antimicrobial activity of nisin, Na-EDTA and pH against gram-negative and gram-positive bacteria. LWT.

[29] Hui G., Liu W., Feng H., Li J., Gao Y. (2016). Effects of chitosan combined with nisin treatment on storage quality of large yellow croaker (Pseudosciaena crocea). Food Chem..

[30] Bonilla J., Fortunati E., Vargas M., Chiralt A., Kenny J.M. (2013). Effects of chitosan on the physicochemical and antimicrobial properties of PLA films. J. Food Eng..

[31] Meira S.M.M., Zehetmeyer G., Jardim A.I., Scheibel J.M., De Oliveira R.V.B., Brandelli A. (2014). Polypropylene/Montmorillonite Nanocomposites Containing Nisin as Antimicrobial Food Packaging. Food Bioprocess. Technol..

[32] Martău G.A., Mihai M., Vodnar D.C. (2019). The use of chitosan, alginate, and pectin in the biomedical and food sector—biocompatibility, bioadhesiveness, and biodegradability. Polymers.

[33] Agriopoulou S., Stamatelopoulou E., Varzakas T. (2020). Advances in Occurrence, Importance, and Mycotoxin Control Strategies: Prevention and Detoxification in Foods. Foods.

[34] Wang X., Yong H., Gao L., Li L., Jin M., Liu J. (2019). Preparation and characterization of antioxidant and pH-sensitive films based on chitosan and black soybean seed coat extract. Food Hydrocoll..

[35] European Commission (1997). Commission Directive 97/48/EC of 29 July 1997 amending for the second time Council Directive 82/711/EEC laying down the basic rules necessary for testing migration of the constituents of plastic materials and articles intended to come into contact with foodstuffs (Text with EEA relevance). Off. J. Eur. Comm..

[36] European Commission (2011). Commission Regulation (EU) No 10/2011 of 14 January 2011 on plastic materials and articles intended to come into contact with food. Off. J. Eur. Union.

[37] Vasile C. (2018). Polymeric Nanocomposites and Nanocoatings for Food Packaging: A Review. Materials.

[38] Castro-Rosas J., Ferreira-Grosso C.R., Gómez-Aldapa C.A., Rangel-Vargas E., Rodríguez-Marín M.L., Guzmán-Ortiz F.A., Falfan-Cortes R.N. (2017). Recent advances in microencapsulation of natural sources of antimicrobial compounds used in food—A review. Food Res. Int..

[39] Salmaso S., Elvassore N., Bertucco A., Lante A., Caliceti P. (2004). Nisin-loaded poly-l-lactide nano-particles produced by CO2 anti-solvent precipitation for sustained antimicrobial activity. Int. J. Pharm..

[40] Shahbazi Y., Shavisi N. (2018). A novel active food packaging film for shelf-life extension of minced beef meat. J. Food Saf..

[41] Sharifian S., Zakipour E., Mortazavi M.S., Arshadi A. (2011). Quality Assessment of Tiger Tooth Croaker (Otolithes ruber) During Ice Storage. Int. J. Food Prop..

[42] Chomnawang C., Nantachai K., Yongsawatdigul J., Thawornchinsombut S., Tungkawachara S. (2007). Chemical and biochemical changes of hybrid catfish fillet stored at 4 °C and its gel properties. Food Chem..

[43] Sun L., Sun J., Liu D., Fu M., Yang X., Guo Y. (2018). The preservative effects of chitosan film incorporated with thinned young apple polyphenols on the quality of grass carp (Ctenopharyngodon idellus) fillets during cold storage: Correlation between the preservative effects and the active properties of the film. Food Packag. Shelf Life.

[44] Eghbal N., Chihib N.-E., Gharsallaoui A. (2020). Nisin. Antimicrobials in Food.

[45] Belfiore C., Castellano P., Vignolo G. (2007). Reduction of Escherichia coli population following treatment with bacteriocins from lactic acid bacteria and chelators. Food Microbiol..

[46] Zimet P., Mombrú Á.W., Faccio R., Brugnini G., Miraballes I., Rufo C., Pardo H. (2018). Optimization and characterization of nisin-loaded alginate-chitosan nanoparticles with antimicrobial activity in lean beef. LWT.

[47] Kim S., Becattini S., Moody T.U., Shliaha P.V., Littmann E.R., Seok R., Gjonbalaj M., Eaton V., Fontana E., Amoretti L. (2019). Microbiota-derived lantibiotic restores resistance against vancomycin-resistant Enterococcus. Nat. Cell Biol..

[48] Bhatia S., Bharti A. (2015). Evaluating the antimicrobial activity of Nisin, Lysozyme and Ethylenediaminetetraacetate incorporated in starch based active food packaging film. J. Food Sci. Technol..

[49] Divsalar E., Tajik H., Moradi M., Forough M., Lotfi M., Kuswandi B. (2018). Characterization of cellulosic paper coated with chitosan-zinc oxide nanocomposite containing nisin and its application in packaging of UF cheese. Int. J. Biol. Macromol..

